# Beyond single-modality screening: toward an EEG–IoT dual-stream framework for early detection of mild cognitive impairment in community-dwelling older adults

**DOI:** 10.3389/fnagi.2026.1877841

**Published:** 2026-07-01

**Authors:** Jingyi Lin, Kaiian Kuok, Wengioi Mio, Caiyi Tan, Puikei Tou, Chonin Cheang

**Affiliations:** 1 Macau Society for Health Economics, Macau, Macau SAR, China; 2 Macau Yinkui Hospital, Macau, Macau SAR, China

**Keywords:** cognitive aging, community screening, digital biomarkers, dual-stream architecture, mild cognitive impairment, multimodal fusion, smart home IoT, wearable EEG

## 1 Introduction

Globally, approximately 55 million people live with dementia, a figure projected to exceed 150 million by 2050 (World Health Organization, 2023). Mild cognitive impairment, the transitional state between normal aging and dementia, affects roughly 17.9% (95% CI: 16.6%−19.2%) of community-dwelling adults aged 65 years and older, with annual conversion rates to dementia ranging from 11.6% to 19.1% (Song et al., 2023). The 2024 Lancet Commission on dementia prevention identified 14 modifiable risk factors collectively attributable to approximately 45% of global cases (Livingston et al., 2024), reinforcing that earlier detection followed by targeted risk-factor modification can meaningfully alter disease trajectories. Yet, early detection at scale remains elusive.

The standard tools of community cognitive screening, the Mini-Mental State Examination (MMSE) and the Montreal Cognitive Assessment (MoCA), are single-timepoint, rater-administered instruments with well-documented limitations. They require in-person attendance, trained administration, and patient cooperation; they are susceptible to practice effects on serial testing; and, importantly, they capture a narrow snapshot of cognition rather than a longitudinal behavioral signal (Nasreddine et al., 2005; Bahreini and Abdi, 2026; Cepukaityte et al., 2024). In community settings across Asia and other regions with aging populations, these structural barriers translate directly into missed diagnoses at the pre-dementia stage when intervention matters most (Huang et al., 2024; Wang et al., 2023).

Two technology streams have emerged as candidates for scalable, ecologically valid MCI screening: wearable electroencephalography (EEG) and smart-home Internet-of-Things (IoT) passive sensing (Cornacchia et al., 2025). Each captures a distinct dimension of cognitive status. Wearable EEG records moment-to-moment neurophysiological dynamics (spectral power ratios, event-related potentials, and cortical connectivity) that directly reflect the synaptic and oscillatory changes underlying MCI pathophysiology (He et al., 2026; Cai et al., 2024). Smart-home IoT networks monitor 24-h behavioral phenotypes (gait variability, nocturnal restlessness, medication adherence, speech cadence), providing ecological data that clinic visits simply cannot replicate (Rawtaer et al., 2020; Lazarou et al., 2024). However, a growing body of evidence reveals that deploying either modality in isolation imposes fundamental ceilings on detection accuracy. We propose that a time-aligned dual-stream architecture, integrating these two data layers, represents the next-generation path to community MCI screening.

## 2 The promise and limits of wearable EEG

Wearable EEG systems, dry-electrode headbands and caps suitable for home deployment, offer a compelling neurophysiological window into the MCI brain. The theta-to-alpha power ratio and reductions in high-frequency gamma complexity are among the most replicated EEG biomarkers of early cognitive decline (He et al., 2026; Meghdadi et al., 2021; Rutkowski et al., 2021, 2023). Frontal and parieto-occipital channels are particularly informative, as posterior alpha slowing and prefrontal theta elevation track well with episodic memory and executive function deficits characteristic of amnestic MCI (Meghdadi et al., 2021; Patro et al., 2025; Li et al., 2024). A multimodal classification framework combining EEG spectral features with speech interaction achieved accuracy of 89.8% in a Chinese cohort of 86 participants using a VR-wearable protocol (Wu et al., 2023), illustrating the potential when task design is carefully optimized (Liang, 2024).

The 2026 systematic review published in *npj Digital Medicine*, the most comprehensive evaluation of its kind, covering 21 studies and 16 distinct wearable EEG devices, confirmed accuracy spanning an extraordinary 46%−95% range (He et al., 2026). The authors conducted a system-level analysis identifying seven critical factors that modulate this variance: channel density, electrode placement, task design, signal preprocessing, feature extraction strategy, classifier type, and, most pertinently, multimodal integration (He et al., 2026). The review explicitly identified multimodal integration as the factor with the most underexploited potential, while simultaneously noting that fewer than a third of the studies included any non-EEG data source. We regard this finding as the central gap to which the present Opinion responds.

Why does single-modality wearable EEG fall short? Three mechanisms dominate. First, real-world EEG recordings in the home are susceptible to movement, environmental, and electrode-contact artifacts that current adaptive preprocessing pipelines mitigate imperfectly (He et al., 2026; Wu et al., 2023). Second, spectral biomarkers such as the theta/alpha ratio are sensitive to acute modulators (fatigue, anxiety, medication, and circadian phase) that are indistinguishable from early cognitive decline without concurrent behavioral context (Meghdadi et al., 2021). Third, validated EEG tasks designed for clinic settings often require active participant engagement that reduces sustained compliance in older adults (Wilson et al., 2025). The result is a technically sophisticated but contextually ungrounded measurement: a neural signal that may be statistically significant at the group level while generating clinically unacceptable uncertainty at the individual level (He et al., 2026). Addressing this requires an external behavioral anchor, precisely what smart-home IoT provides.

## 3 The promise and limits of smart-home IoT behavioral sensing

Smart-home passive sensing platforms deploy arrays of motion sensors (passive infrared), door and appliance contact sensors, bed and wearable activity bands, and, increasingly, ambient microphones to build continuous behavioral phenotypes of older adults in their natural environment (Rawtaer et al., 2020; Piau et al., 2019; Chung et al., 2024). The behavioral biomarkers most consistently linked to MCI include reduced daytime activity, increased sleep fragmentation, greater gait speed variability, higher medication-miss rates, and slowing of routine task completion (Piau et al., 2019; Aleid et al., 2023; Giaretta et al., 2022; Shih et al., 2024). A Singapore-based feasibility study demonstrated that IoT-derived behavioral metrics distinguished MCI from healthy cognition in 49 community-dwelling older adults, with the sensor system achieving acceptability rates above 80% (Rawtaer et al., 2020). A 2024 cross-sectional study using a purpose-built smart home environment found that activities-of-daily-living monitoring separated healthy controls, subjective cognitive decline, and MCI groups with discriminant validity (Lazarou et al., 2024). A 2026 systematic review of wearable and digital biomarker technologies for early cognitive impairment detection, spanning 49 studies and over 200,000 participants, confirmed that behavioral digital biomarkers reliably stratify early cognitive risk but remain predominantly confined to the behavioral layer (Cejudo et al., 2026).

Longitudinal sensor cohorts are advancing rapidly. The SINEW cohort (Singapore, *n* = 138 as of June 2025) is installing multi-sensor home systems including infrared, door contact, bed, medication-box, and wearable activity devices to develop machine-learning models for predicting MCI and frailty transitions, with planned recruitment through 2030 (Moon et al., 2026). Remote monitoring frameworks reviewed in a 2025 *JMIR Aging* analysis further confirm that IoT-based systems can monitor AD/ADRD patients' safety, medication adherence, and behavioral health parameters in the home setting (Shaik, 2025). Despite this progress, a fundamental limitation persists: behavioral changes (gait slowing, sleep disruption, social withdrawal) arise from multiple causes beyond cognitive decline, including depression, pain, polypharmacy side effects, and intercurrent illness (Piau et al., 2019; Cejudo et al., 2026). Without a neurophysiological reference signal, IoT behavioral systems cannot distinguish signal from noise at the individual level. High false-positive rates translate into unnecessary downstream clinical burden and participant anxiety. What IoT behavioral sensing lacks is precisely what wearable EEG provides: a moment-to-moment physiological ground truth.

## 4 A dual-stream architecture: time-aligned neural and behavioral data

**Figure 1:**
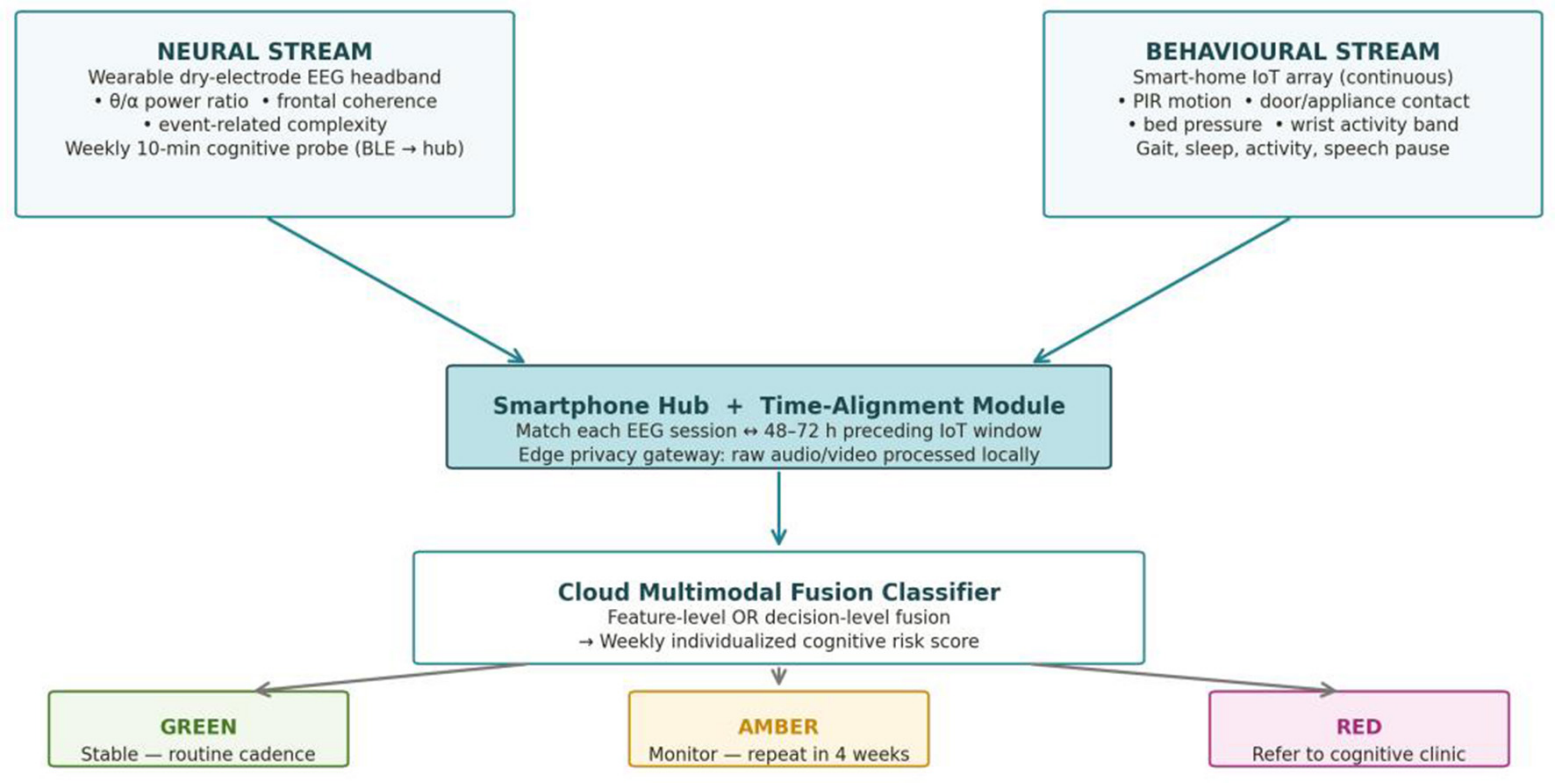
Conceptual architecture of the EEG–IoT dual-stream MCI screening framework. The figure illustrates a two-layer system operating in a community-dwelling older adult's home environment. The left stream (neural layer) depicts a wearable dry-electrode EEG headband transmitting theta/alpha power ratios, frontal coherence, and event-related complexity metrics via Bluetooth to a smartphone hub during brief weekly cognitive probe sessions. The right stream (behavioral layer) depicts an array of smart-home sensors (passive infrared motion sensors, door/appliance contact sensors, a bed pressure sensor, and a wrist activity band) continuously streaming gait cadence, sleep fragmentation, activity distribution, and speech pause frequency to the same hub. At the hub, a time-alignment module matches each EEG session to a 48–72-h pre-session IoT behavioral window; the concatenated feature vector is transmitted to a secure cloud server for feature-level or decision-level fusion classification, producing a weekly individualized cognitive risk score. Output feeds a stratified alert system: green (stable), amber (monitor, repeat in four weeks), and red (refer to primary care cognitive clinic). A privacy gateway at the edge ensures raw audio and video are processed locally and only anonymized features are transmitted. EEG, electroencephalography; IoT, Internet of Things; PIR, passive infrared; BLE, Bluetooth Low Energy; θ/α, theta-to-alpha power ratio. Solid arrows denote data flow. The edge privacy gateway ensures only anonymized feature vectors leave the home; raw audio and video are processed locally and never transmitted.

We propose a dual-stream MCI screening architecture in which a wearable EEG neural layer and a smart-home IoT behavioral layer are recorded concurrently, synchronized on a common timestamp, and fused through a multimodal classifier. The framework is illustrated conceptually in Figure 1.

### 4.1 Neural stream

The EEG stream captures theta/alpha power ratios, frontal beta coherence, and event-related complexity metrics during brief, elderly-friendly cognitive probe tasks performed at home (for example, a 10-min smartphone-guided memory or naming task administered three times weekly). Device candidates include single-band dry-electrode headbands (2–4 frontal-parietal channels) that achieve acceptable accuracy while minimizing setup burden for older users (He et al., 2026; Wilson et al., 2025). On-device preprocessing filters motion artifacts, and a cloud-based adaptive pipeline applies subject-specific thresholding to flag artifact-contaminated segments.

### 4.2 Behavioral stream

The IoT stream operates continuously. Passive infrared sensors log activity distributions, sleep onset and offset, nocturnal restlessness counts, and inter-room movement trajectories. Smart plugs track kitchen appliance usage as a proxy for meal preparation regularity. A bed sensor extracts sleep-stage proxies via pressure variability. Wearable activity bands contribute step counts, gait cadence, and heart rate variability. Ambient passive voice capture (with appropriate consent and edge-processing for privacy) extracts speech rate variability and pause frequency, both established digital speech biomarkers of early cognitive change (Ding et al., 2022; Yang et al., 2023). The composite IoT feature vector is updated daily.

### 4.3 Fusion and classification

Time alignment is the architecturally critical step. Each EEG recording session is matched to an IoT behavioral window spanning the preceding 48–72 h, capturing the behavioral state in which the neural measurement is embedded. Feature-level fusion concatenates the EEG spectral vector with the IoT behavioral feature vector; a gradient-boosted or transformer-based ensemble classifier then outputs an individualized risk score (Asl and Rabie, 2025; Liu et al., 2025). Alternatively, decision-level fusion (combining independent EEG-derived and IoT-derived probability scores via a learned weight matrix) offers greater interpretability and graceful degradation when one stream is temporarily unavailable. A 2025 multimodal machine-learning study integrating structural MRI, accelerometry, polygenic risk scores, and lifestyle data for early neurodegenerative disease prediction achieved a best AUC of 0.819, with the full multimodal model outperforming all single-modality and partial-fusion baselines (the model excluding MRI parameters fell to AUC 0.688), demonstrating the consistent additive value of behavioral-sensor data when fused with biological signal (Li et al., 2025; Agostinho et al., 2024; Cirincione et al., 2024).

### 4.4 Technical challenges

Three challenges must be addressed for real-world deployment. First, sampling rate heterogeneity: EEG streams at 250–500 Hz while IoT behavioral data is event-driven or sampled at 1 Hz or less; temporal window selection and feature aggregation protocols require careful standardization (Wu et al., 2023). Second, older-adult compliance: head-worn EEG devices remain less accepted than wrist-worn devices (Wilson et al., 2025); compliance monitoring and simplified onboarding protocols (guided by patient-centered design and caregiver involvement) are essential. Third, feature stability: both EEG and behavioral metrics fluctuate with non-cognitive factors; the fusion model must incorporate day-of-week patterns, health event flags, and medication records to avoid confounding (Piau et al., 2019; Li et al., 2025).

The wearable devices in elderly chronic disease management literature from mainland Chinese institutions underscores that barriers and facilitators to IoT adoption in this population are well-characterized, including the importance of low setup complexity, caregiver involvement, and demonstration of tangible benefit (Zhang et al., 2025). These insights should inform dual-stream device design from inception.

## 5 Discussion: from concept to community deployment

The dual-stream architecture we propose aligns with the broader push toward continuous, ecologically valid brain health monitoring articulated in the 2026 passive digital biomarker study (Matias et al., 2026) and the 2026 AI-wearables systematic review (Cejudo et al., 2026). The most plausible near-term target population for a pilot is adults aged 65 years and older with subjective cognitive complaints not yet meeting formal MCI criteria, those at highest progression risk but for whom current screening delivers the least actionable information (Livingston et al., 2024; Zolfaghari et al., 2024).

We draw particular attention to the Greater Bay Area (GBA: Guangdong, Hong Kong, and Macau) as a strategically suitable site for a first feasibility cohort. Healthcare integration policies in the Hengqin Guangdong-Macau In-Depth Cooperation Zone, active wearable neurotechnology programs at Macau University of Science and Technology (Song et al., 2023), and established community cognitive screening initiatives in Hong Kong (Chan et al., 2020) create a favorable institutional environment (Zang et al., 2025). A China national consensus on rapid screening for prodromal Alzheimer's disease further underscores readiness for technology-supported early detection pathways (Huang et al., 2024).

Ethical implementation requires attention to three principles. First, consent for passive behavioral monitoring must specify data collection scope, storage, and the right to withdraw; simplified consent procedures adapted for older adults with incipient cognitive changes are mandatory (Rawtaer et al., 2020). Second, data minimization: only features required for classification should be transmitted; raw audio and video must remain on-device. Third, algorithmic fairness: training cohorts must reflect the educational and linguistic diversity of GBA communities (He et al., 2026).

Regulatory pathways remain relevant. Establishing a validated performance benchmark (sensitivity ≥80% and specificity ≥75% against clinician-confirmed MCI) would support regulatory submissions in both China and Hong Kong (Cejudo et al., 2026). The 2024 Lancet Commission's framing of MCI screening as preventive rather than diagnostic may further ease regulatory categorization (Livingston et al., 2024).

We acknowledge that the dual-stream framework presented here is conceptual and requires prospective validation. Key unknowns include the minimum EEG session frequency for reliable biomarker estimation, the optimal IoT behavioral window length for fusion, and the marginal accuracy gain from adding the neural stream to an IoT-only model. We call on researchers working across wearable neurophysiology, digital health, and geriatric medicine to design prospective cohort studies that formally test these parameters.
